# Examining recruitment feasibility and related outcomes in adults post-stroke

**DOI:** 10.1186/s40814-020-00696-w

**Published:** 2020-10-24

**Authors:** Erin C King, Megan Doherty, Daniel Corcos, Mary Ellen Stoykov

**Affiliations:** 1 Interdepartmental Neuroscience Program, Northwestern University, 645 N Michigan Ave, STE 1100, Chicago, IL 60611 USA; 2 Swedish Medical Center, Englewood, Colorado USA; 3 Northwestern University, Chicago, Illinois USA; 4 Shirley Ryan Ability Lab, Chicago, Illinois USA

## Abstract

**Background:**

There are limited effective and evidence-based interventions for upper extremity hemiparesis post-stroke. To prepare for an RCT and minimize misuse of resources, there is value in conducting a feasibility study.

**Objective:**

To examine the feasibility of recruitment and other related outcomes for an intense upper limb intervention.

**Methodology:**

Feasibility outcomes included retention, adherence, accrual rate, sample characteristics, and identification of productive recruitment methods. Other outcomes included satisfaction with the study, fidelity, and equipoise of both staff and participants.

**Results:**

Participants were enrolled at a rate of 1.33 per month. The recruitment timeline had to be extended by 4 months, to meet the target of 16 randomized participants. Staggered recruitment was the most successful strategy. We found that following up with individuals who missed initial appointments prior to study enrollment led to decreased adherence.

**Conclusion:**

It is feasible to recruit and retain post-stroke participants for an intense intervention study.

**Trial registration:**

NCT02277028

## Key messages regarding feasibility

- What uncertainties existed regarding the feasibility?
  - The feasibility of recruiting enough participants in a 1-year timeline.
  - The feasibility of recruiting a representative sample of the population surrounding the medical center at which the study took place.
  - The feasibility of older adults adhering to an intensive rehabilitation protocol.
- What are the key feasibility findings?

- It is feasible to recruit and retain post-stroke participants for an intense intervention study.

- What are the implications of the feasibility findings for the design of the main study?
  - The findings of this study provide important information about recruitment and adherence to an intensive upper limb rehabilitation RCT in pursuit of developing high impact, functional interventions for individuals affected by stroke.

## Background

Recovery of motor function in the affected upper extremity (UE) is a predictor of an individual’s ability to live independently after a stroke [1]. It is therefore critical to develop therapeutic interventions which promote UE recovery and improve performance in daily activities. Constraint-induced movement therapy is the intervention for upper limb hemiparesis with the most evidence for therapeutic efficacy. However, the strict inclusion criteria render 80% of individuals with UE hemiparesis ineligible for the treatment [2, 3].

Currently, effective therapeutic interventions for UE hemiparesis are limited due, in part, to a lack of rigorous research. Randomized controlled trials (RCTs) provide the most reliable evidence for health care interventions; however, they are costly and labor intensive. Thus, there is value in first conducting feasibility studies to assess the intervention and discover any factors that may complicate or compromise a larger clinical trial. In doing so, researchers can avoid wasting funding resources and participants’ time [4]. It is well documented that recruitment to clinical trials is a major obstacle to trial success [5]. Conducting a feasibility study offers a way of assessing the usefulness of specific recruitment strategies, determining the timespan needed to recruit the sample size, and estimating a budget for a future study [6].

Our feasibility study was designed to investigate outcomes related to recruitment including adherence, retention, accrual rate, characteristics of the sample, and reliable recruitment methods. Although there are significant challenges posed by recruitment, studies aimed at examining the efficacy of specific recruitment strategies in clinical trials are scarce [7]. This study analyzes the strength of each recruitment source and discusses participant flow thereafter in an effort to share effective strategies for recruitment into an RCT. Characteristics of the sample were also documented.

According to Robiner [8], adherence in a clinical trial is the degree to which behavior of study participants corresponds to the study protocol. If participants do not adhere, results of the clinical trial can be inconclusive at best or, in worst case, invalid [6, 9, 10]. Adherence, in our study, was defined as attendance and participation consistent with the protocol at all 15 treatment (Tx) sessions as well as completing the final session (Tx day 15) no later than 8 weeks post Tx day 1. Retention was measured at both post-treatment and follow-up.

Other study-related outcomes included equipoise, fidelity, and participant satisfaction.

Equipoise, the existence of uncertainty that one intervention is more effective than another, should be maintained by the principal investigator (PI), study staff, and study participants [11]. Maintaining equipoise is important to attenuating bias and promoting scientific credibility. Equipoise for participants as well as research staff was determined, respectively, by polling during and at the end of the study.

Fidelity is a methodological process used by investigators to track and determine the extent to which evaluation and treatment procedures are delivered by the study staff as intended [12]. Rater fidelity requires training in all evaluation tools and subsequent monitoring by the investigator to ensure raters continue to administer the assessments according to previously established guidelines. The intervention must be administered as indicated in the intervention study protocol in order to conclude that any outcomes are a true reflection of intervention design [13]. Maintaining fidelity improves internal validity, increases power, and allows for replication of the study [14]. Both treatment therapists and the masked rater had to pass fidelity checks prior to and during the study.

This study examined the feasibility of recruiting post-stroke participants and collected study-related outcomes including (1) equipoise of study participants and staff, (2) fidelity of procedures by the study staff, and (3) participant satisfaction. Feasibility outcomes included adherence, retention, accrual rate, identification of characteristics of sample, and determination of productive recruitment methods. Patient satisfaction with the treatment was measured by the Canadian Occupational Performance Measure (COPM) [15]. We hypothesized that we could meet our recruitment goal in 8 months and that our retention and adherence rate would be 90%. We also hypothesized that participants, regardless of group assignment, would be satisfied as measured by the COPM satisfaction scale. The latter measures satisfaction with occupational performance and not study satisfaction per se. However, satisfaction with occupational performance is essential to overall satisfaction for an occupational therapy study. Though this paper will not discuss efficacy, the intervention outcome measures used included the Chedoke Arm and Hand Activity Index (CAHAI 9) and the Fugl Meyer Upper Extremity Test of Function (FMUE).

## Methods

### Design

Institutional review board (IRB) approval was granted by Rush University Medical Center to conduct this study. All potential participants who visited the lab signed a consent form according to the declaration of Helsinki. This was a single masked, parallel group, randomized controlled feasibility trial to examine recruitment feasibility, adherence, participant satisfaction, and equipoise of an intensive arm intervention for older adults.

### Participants

Participants were informed of the study’s risks and benefits and consented by the PI. Eligibility criteria included (a) no orthopedic conditions of the contralateral or ipsilateral wrist, (b) 55 years of age and over, (c) at least 6 months post-stroke, (d) unilateral stroke, and (e) an FMUE score between 23 and 38 (inclusive). Participants were deemed ineligible if they had contraindications to transcranial magnetic stimulation (TMS) including (a) metal implants of any size in the head or neck area; (b) history of epilepsy, seizures, or convulsions; (c) previous head trauma or concussion with loss of consciousness; (d) cochlear implants; (e) history of ongoing headaches; and (f) presence of a pacemaker.

Eligible participants were assigned to the bilateral motor priming (BMP) group or the health care education (HCE) group using stratified randomization via two computer-generated number lists. Groups were stratified by their level of impairment, determined by the participant’s initial score on the FMUE. Each participant, within his or her designated group, had an equal chance of being randomized into the BMP group or the HCE group.

### Intervention

The intervention focused on reducing arm and hand impairment and disability using BMP, which was delivered prior to a task-specific training (TST) protocol. The latter TST protocol has shown evidence of efficacy in a previous clinical trial [16, 17]. Other studies have demonstrated the potential that BMP holds for motor recovery by utilizing symmetric movements which prime the brain for subsequent motor training [18–20]. The active comparator group received health care education prior to the same TST protocol. The HCE intervention was delivered in the form of a computerized Jeopardy game. Participants were instructed to use their affected hand as much as possible for computer operation. The information for the Jeopardy game was taken from the American Heart Association website.

Priming and TST were led by an occupational therapist. All treating therapists were familiarized with study protocol and standardized according to a qualifying checklist. Following 15 min of either BMP or HCE, participants received 45 min of TST. This was followed by a 1-h break. Participants returned and completed a similarly structured additional hour of therapy (Table 1). The TST protocol was divided into two, 45-min sessions. The first session included reaching and prehension tasks, while the second session focused on the participant-identified activities of daily living (ADL) via the COPM.

**Table 1:** Priming and intervention schedule

HCE group	Time	BMP group
HCE	15 min	BMP
Grasp and reach TST	45 min	Grasp and reach TST
Break	1 h	Break
HCE	15 min	BMP
Occupation-based TST	45 min	Occupation-based TST

### Feasibility outcomes

#### Adherence

Our initial plan was for participants to complete the study in 5 weeks. This would require that the participants have a 3-h appointment (2 h of training with a 1-h break). However, due to a variety of issues, participants could not always keep up with the schedule, and, thus, we extended their schedule as needed. If participants took longer than 6 weeks, we allowed them to stay in the study in order to examine the response to treatment. Adherence metrics included the attendance at treatment sessions and the number of weeks it took participants to complete the 15 sessions.

We counted the number of days from the first to the last day of intervention and divided this number by seven to calculate total number of weeks to complete the treatment protocol (Tx timespan). The number of sessions per week was calculated by dividing 15 (total # of sessions in protocol) by TX timespan. We then multiplied this number by 2 to determine the total number of treatment hours per week.

TX hours per week = [15 ÷ (# days from first day to final TX day ÷ 7)] × 2

#### Retention

Percent retention was measured at post and follow-up by dividing the number of participants who were present at these time points by the number of individuals who were enrolled and began the training protocol.

#### Accrual rate

In order to determine the difference between projected and actual recruitment and enrollment, the number of participants enrolled each month was compared to the target enrollment timeline. Accrual rate was determined by the total number of enrolled participants divided by the number of months recruitment occurred.

#### Sample demographics

We sought to recruit a representative sample of the population surrounding the medical center at which the study took place. Target recruitment goals for race and sex were formulated based on demographics of adjacent neighborhoods, reported as 39% Hispanic, 32% Black, 24% White, and 5% other [21]. Characteristics of the sample were measured using frequencies and descriptive statistics.

#### Productive recruitment methods

Recruitment methods included an IRB-approved flyer, presentations to outpatient occupational therapists, contacting stroke survivors known to the PI, engaging neurologists from the medical center, and contacting outside stroke rehabilitation investigators. Methods by which participants were referred to the study were tracked.

Participants contacted the PI by phone, or the study team was notified of their interest via a referral. During the initial phone screen, the PI informed the potential participants of the inclusion and exclusion criteria and described the study. If the potential participant met the age criteria, reported that he/she had none of the exclusion criteria, and expressed interest in joining the study, a lab screening was conducted by a masked assessor using the FMUE. Throughout the screening process, reasons for ineligibility were recorded.

### Study related outcomes

#### Equipoise

Equipoise was tracked through regular polling of all research staff. Bi-monthly, the PI asked staff if they had any indication if one group was benefitting from treatment more than another. The masked assessor was randomly polled to ascertain if she was aware of any participant’s group randomization. The participants were queried at the end of the study if they, at any point during the study, were aware of being assigned to either the experimental or control group.

#### Fidelity

To ensure fidelity to assessment procedures including the CAHAI 9 and the FMUE, the PI evaluated the masked rater during administration of the tests and completed a fidelity checklist every 6 months. A passing score consisted of 95% or better on both assessments was required. To ensure the TST intervention protocol was administered according to principles of the protocol, the PI observed the treatment occupational therapist administering the protocol every 6 months. The PI completed a checklist documenting intervention fidelity, and a minimum passing score of 90% was required.

#### Participant satisfaction

Participant satisfaction was measured by satisfaction scores from the COPM, a reliable source for satisfaction scores in stroke patients [22]. The COPM was administered at the first session to guide treatment. The COPM was re-administered at the post-treatment evaluation. At post-treatment, we also queried participants to determine if they were satisfied with the study via a short, written questionnaire.

#### Statistical analysis

Analysis of intervention outcome data was conducted using SPSS version 19 and Microsoft Excel 2010. Descriptive statistics were used to determine frequency of recruitment sources, demographic information, retention rates, adherence, and equipoise. A mixed, two-way repeated measures ANOVA analyzed the COPM satisfaction scores pre-intervention versus post-intervention.

## Results

### Participants

**Fig. 1:**
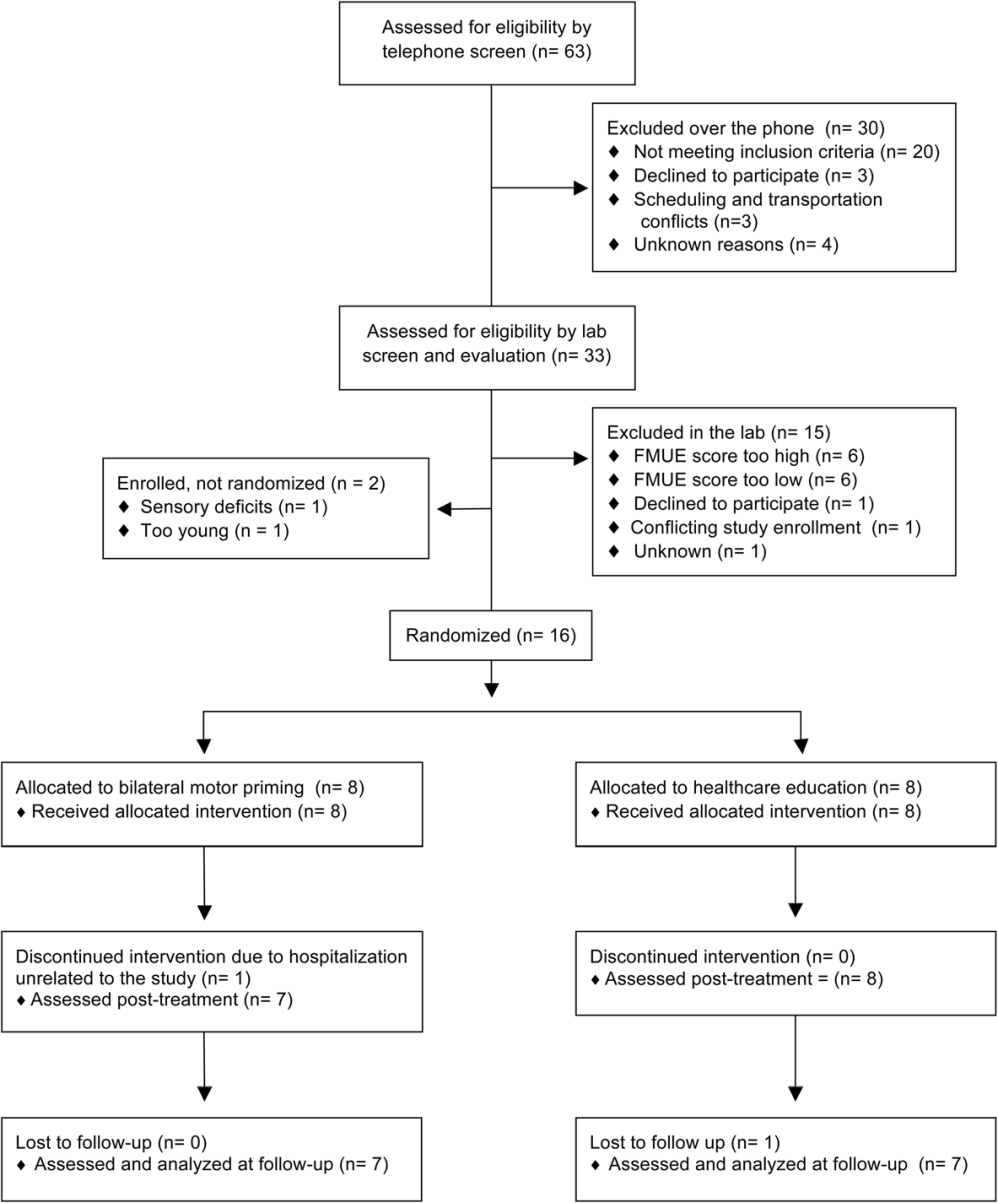
CONSORT flow diagram. Courtesy of IOS Press, Stoykov et al. [23], Restorative Neurology and Neuroscience

Figure 1 illustrates the study flow diagram. Sixty-three individuals from Chicago and the surrounding neighborhoods were contacted and underwent an initial phone screen. Thirty individuals were excluded over the phone. Thirty-three participants were further evaluated for eligibility in the laboratory using the Fugl-Meyer Upper Extremity Test. Twelve individuals that came for the lab screen were ineligible due to their Fugl-Meyer score. One individual was ineligible because she was enrolled in another study, one declined to participate, and one individual was not enrolled for unknown reasons. Two participants were found to be ineligible after enrollment. One of the participants was deemed ineligible due to complete sensory loss that was not determined at screening. The second participant was ineligible due to age. Though enrolled, they were not randomized. Their feasibility data is included here. They were not included in the analysis of primary and secondary outcomes, which are reported elsewhere [23].

A final total of 16 enrolled participants were stratified by FMUE scores and then randomized as previously described. During the course of the study, one participant was hospitalized for unrelated reasons. An additional participant was lost to follow-up due to a comorbidity. The recruitment period was from July 2014 to July 2015. The last follow-up session was in February of 2016.

### Adherence

The median treatment timespan (# of days between Tx day 1 and 15 ÷ 7) was 6.05 weeks (IQR 2.86), and the median treatment sessions per week were 2.48 (IQR = 0.74). Median treatment hours per week were 4.95 (IQR = 2.09). We set adherence as the number of completed training sessions in 8 weeks or less resulting in a final adherence rate of 95%.

### Retention

Our retention rate for post-treatment was 94%. During the course of the study, one participant was hospitalized for unrelated reasons, and we were unable to obtain his post-treatment evaluation. Another participant finished the treatment and had a post-treatment evaluation. However, he refused a follow-up appointment. This resulted in a retention rate of 87% at follow-up, which was below our hypothesized rate of 90%.

### Accrual rate

**Fig. 2:**
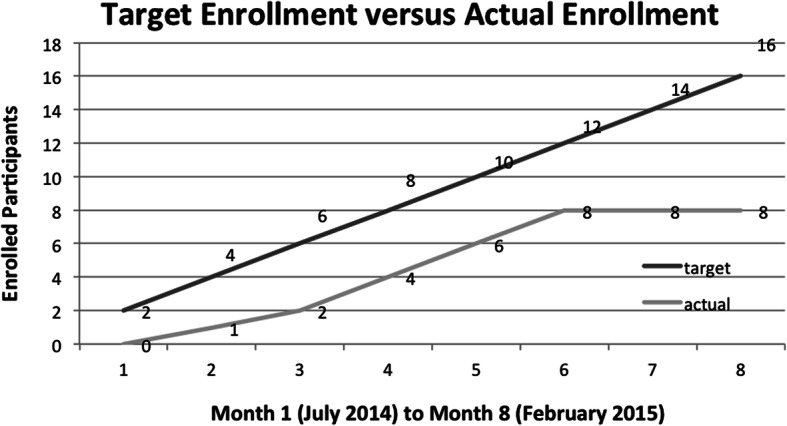
Original 8-month recruitment accrual timeline

**Fig. 3:**
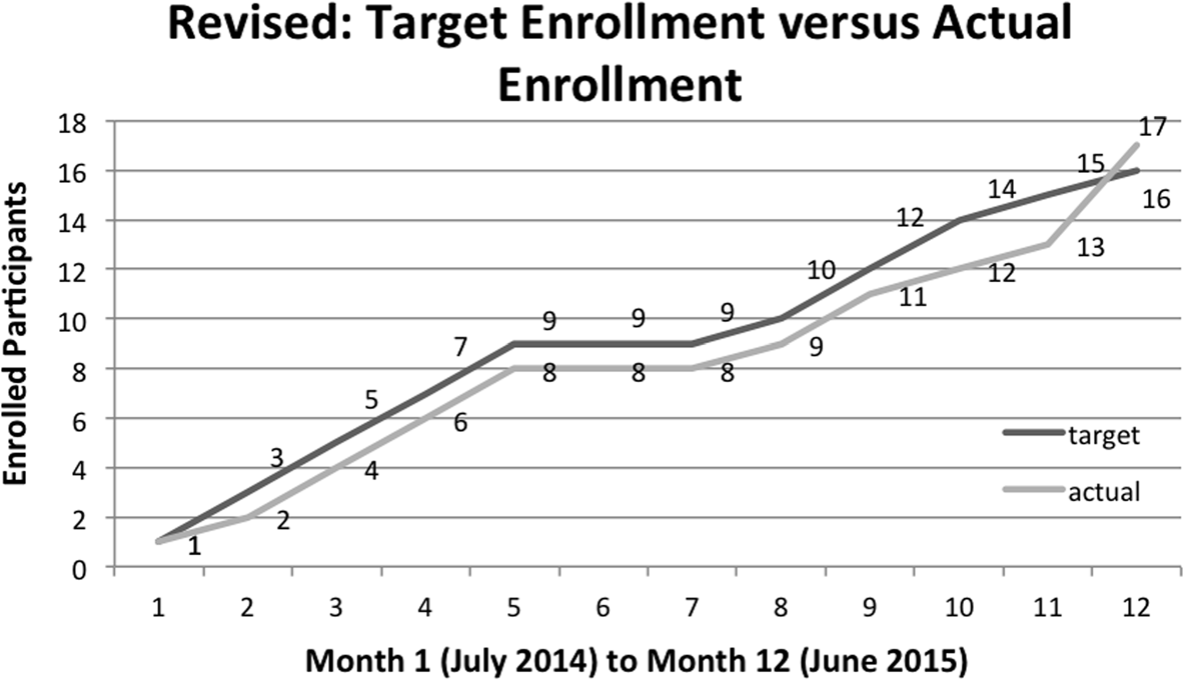
Twelve-month revised recruitment accrual timeline

**Fig. 4:**
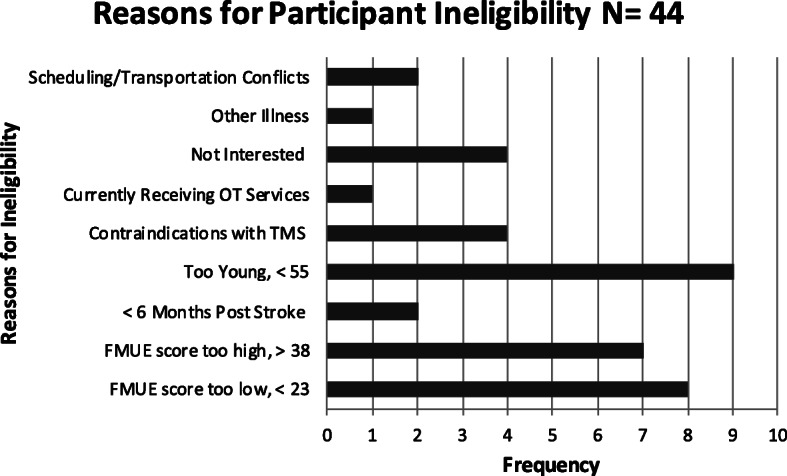
Frequency of reasons for participant ineligibility

The timeline for completing the study needed to be modified based on a recruitment lag during winter months. The original timeline for 8 months was therefore extended to 12 months (see Figs. 2 and 3). We were able to recruit the targeted number of participants in 12 months with a final accrual rate of 1.33 participants per month. The primary reason for ineligibility was age, followed by severe motor impairment, considered too low level. Additional reasons are illustrated in Fig. 4.

### Sample characteristics

Participants identifying as Asian (2), Black (11) and White (4) were enrolled into the study. Three females and 14 males were enrolled. Demographics of enrolled participants can be found in Table 2.

**Table 2:** Demographics of enrolled participants by treatment group

Characteristics	BMP	HCE
Age, mean (SD)	61 (7.6)	63 (5.21)
Months post-stroke, mean (SD)	62.9 (50.0)	68.13 (51.1)
Baseline FMUE score, mean (SD)	29.2 (4.16)	29 (5.42)
Male	8	6
Female	1	2
Race		
Asian	1	1
Black	5	6
White	3	1

Courtesy of IOS Press, Stoykov et al. [23], Restorative Neurology and Neuroscience

### Productive recruitment methods

**Fig. 5:**
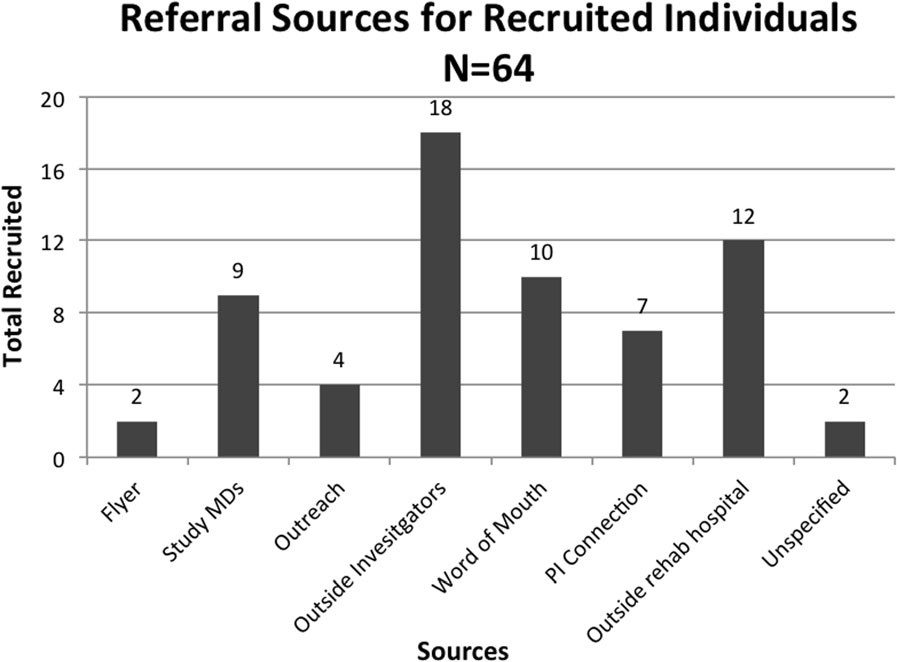
Frequency of recruited individuals by referral source

The single highest yielding recruitment source for screened (*n* = 15) and enrolled (*n* = 6) participants was from an outside investigator conducting a similar study for the lower extremity at a nearby university. Figure 5 illustrates the frequency of recruited individuals by referral sources.

### Equipoise

The treatment therapist and the research assistants (not masked) consistently responded that improvement was due to the TST protocol and the number of repetitions that the participant was able to complete. The masked assessor consistently reported having no knowledge of group assignment; therefore, the single-masked protocol of this study was maintained. Lastly, only one study participant correctly identified group assignment. Others either reported they were unaware of group assignment or they incorrectly identified their group assignment.

### Fidelity

The assessor passed the FMUE and CAHAI fidelity checklist at the initial standardization but was required to retake the FMUE standardization after receiving a score below 95% during the 6-month check-in. A passing score was achieved after review of procedures and repeating standardization. The treating occupational therapist passed the TST protocol fidelity checklist with scores above 90% prior to treating the first study participant and at each six-month check-in.

### Participant satisfaction

**Fig. 6:**
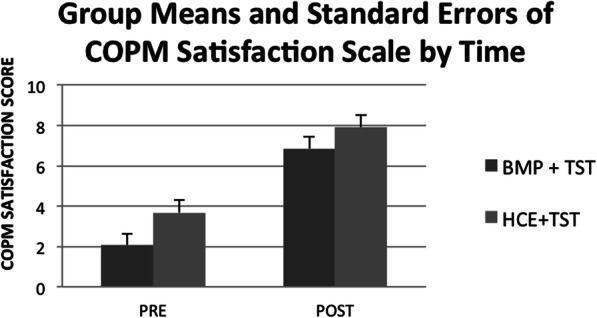
COPM satisfaction scores by group and time

A mixed two-way repeated measures ANOVA with group as the between factor and time (pre/post-intervention) as repeated factor demonstrated a significant effect of time (*F* = 47.8, *p* < .001) but no interaction between group and time. This indicated that both the experimental and the active comparator group had improved satisfaction in their self-selected ADL tasks from pre-intervention to post-intervention (Fig. 6).

## Discussion

This study examined the feasibility of recruiting post-stroke participants to an intensive post-stroke upper limb rehabilitation study. We found it feasible to recruit the goal of 16 participants (1.33 participants per month) within an extended time period of 1 year. Research staff and participant equipoise was maintained. Fidelity of the treatment therapists was satisfactory. The masked rater needed additional review of procedures at the 6-month check in. Participants were highly satisfied.

### Feasibility outcomes

#### Adherence

In an effort to maintain our accrual rate, our team followed up with individuals who either did not show up for the first evaluation or did not return phone calls. We found, however, that this was a poor strategy. Difficulty contacting a specific individual was considered a red flag for low adherence. Ideally, low adherers should be identified prior to randomization. Questions asked during screening may filter out those who are not likely to adhere to the schedule. Possible indications of non-adherers include recent hospitalization, lack of access to reliable transportation, substance abuse issues, possibility of vacation or relocation during study, prior non-adherence in previous studies, and large distance between home and study location [9].

In our study, two low adherers were hospitalized prior to enrollment, and one had consistent issues with transportation. The two individuals (one in each group) consistently missed appointments and both took longer than 8.5 weeks to complete the training due, in part, to cognitive difficulties and other medical problems. Neither participant made improvement on the CAHAI 9 or FMUE. These participants had longer treatment timespans than the other participants (> 8.5 weeks).

Low adherence can reduce the power of the study to detect a treatment effect. In order to attenuate the latter, investigators can do two things: (1) build in realistic adherence rates to the study; and/or (2) identify and weed out non-adherers prior to randomization [9]. In addition to a dropout rate, a non-adherence rate might be estimated based on previous samples. This will increase the size of the sample but also increase the possibility of obtaining meaningful results.

Our initial plan outlined that participants should complete the training over the course of 4 to 5 weeks. We did not anticipate that participants would require more than 6 weeks to complete training. Most of our eligible participants did not want to attend sessions more than 3 days per week on average.

This study provides very preliminary evidence regarding dosage of studies designed to remediate post-stroke impairment. In this study, individuals who completed training in a larger timespan (> 8 weeks to complete 30 h) had an attenuated treatment effect. This is important information for future upper limb training studies. In fact, a timespan greater than 8.0 weeks to complete 30 h of treatment (approximately 3.5 h of treatment per week) was insufficient to remediate motor impairment. Extending the time taken to complete the treatment was not optimal as post-stroke neuroplasticity is dependent on intensity and frequency of treatment. Based on this principle, the efficacy of treatment in this study relied upon the number of hours completed in a set time frame.

#### Retention

Study staff took an active role in maintaining rapport with participants by addressing concerns, respecting cultures and schedules, and becoming familiar with participants’ lives. Participants received a stipend per session to account for time and transportation costs. When necessary, research staff was available to transport participants from the hospital entrance to the research lab. A member of the research team was responsible for contacting participants for session reminders or rescheduling when necessary.

#### Sample characteristics

Contrary to expectations, we had no difficulty with minority recruitment. We concluded that it is certainly feasible to recruit participants from diverse racial and ethnic backgrounds to a stroke intervention study in an urban setting.

We expected to enroll nearly equal numbers of males and females. However, this was not the case, as only 17% of our participants were female. Di Carlo et al. [24] found that being female was a significant predictor of disability at 3 months post-stroke even after controlling for baseline and clinical variables. Women may experience greater difficulty enrolling in clinical trials as compared to men possibly due to disability status. Cultural and social factors may also play a role. Previous research has explored low enrollment of women into drug and cardiovascular clinical trials. Limited research exists to both establish low enrollment of women in to stroke rehabilitation trials and further, identify factors driving low enrollment. Larger studies are needed to determine representation and barriers to recruitment relative to the stroke population.

#### Productive recruitment methods

Due to the competitive nature of doing research in a city rich in academic medical centers, it benefitted our study to utilize staggered recruitment/enrollment strategies. Staggered recruitment techniques require cooperation between different investigators and their respective study staff [9]. When participants completed a study at a nearby institution, they were subsequently referred to our study and vice versa. This investigator also used transcranial magnetic stimulation (TMS) as an evaluation measure. Thus, individuals referred from this source had been previously screened for exclusion based on TMS restrictions. This collaboration yielded 15 potential participants, six of whom were enrolled.

### Other study-related outcome measures

#### Equipoise

We were surprised that the participants were not able to correctly identify whether they were in the experimental or control group. We speculate that, since the treatment protocol was the same for both groups, the participant’s perception of their own improvement was high regardless of group assignment. Indeed, according to the COPM, both groups improved equally on their satisfaction with their self-selected occupational tasks. We maintain that participant equipoise was high for this study.

#### Fidelity

The main treatment therapist was able to maintain fidelity throughout. Although the masked rater passed the initial standardization, her re-standardization was below 95%. This reinforces the importance of frequent fidelity checks for raters.

#### Participant satisfaction

Participants in both groups were highly satisfied as evidenced by the COPM. We received several thank you cards and one participant communicated a desire for additional time in the study.

## Conclusion

This paper describes in detail how feasibility (including adherence, retention, accrual rate, sample characteristics, and recruitment methods) and related outcomes (equipoise, fidelity and satisfaction) were measured in an intensive occupational therapy intervention study with older adult participants. There are few randomized controlled feasibility trials in rehabilitation that discuss recruitment methods. Moreover, there are insufficient number of RCTs within occupational therapy research that demonstrate the necessary rigor in design and methods to achieve reliable outcomes. This manuscript provides important information about planning intensive upper limb rehabilitation feasibility studies in pursuit of developing high impact, functional outcomes for individuals affected by stroke.

## Data Availability

The datasets during and/or analyzed during are the current study available from the corresponding author on reasonable request.

## Abbreviations

UE: Upper extremity
BMP: Bilateral motor priming
HCE: Health care education
TST: Task-specific training
RCT: Randomized controlled trials
FMUE: Fugl Meyer Upper Extremity Test of Function
COPM: Canadian Occupational Performance Measure
CAHAI: Chedoke Arm and Hand Activity Index
Tx: Treatment
PI: Principal investigator
IRB: Institutional review board
TMS: Transcranial magnetic stimulation
ADL: Activity of daily living
